# Influence of Different Methionine Sources on Performance and Slaughter Characteristics of Broilers

**DOI:** 10.3390/ani9110984

**Published:** 2019-11-19

**Authors:** Cristina Ullrich, Marion Langeheine, Ralph Brehm, Venja Taube, Mercedes Rosillo Galera, Karl Rohn, Johanna Popp, Christian Visscher

**Affiliations:** 1 Institute for Animal Nutrition, University of Veterinary Medicine Hannover, Foundation, Bischofsholer Damm 15, D-30173 Hanover, Germany; Cristina.ullrich@tiho-hannover.de; 2 Institute for Anatomy, University of Veterinary Medicine Hannover, Foundation, Bischofsholer Damm 15, D-30173 Hanover, Germany; Marion.langeheine@tiho-hannover.de (M.L.); Ralph.brehm@tiho-hannover.de (R.B.); 3 BEST 3 Gefluegelernaehrung GmbH, Ringstrasse 16, D-27239 Twistringen, Germany; V.taube@best-3.de; 4 University of Granada, Avenida del Hospicio s/n, 18010 Granada, Spain; mercedesrosillo@correo.ugr.es; 5 Institute for Biometry, Epidemiology and Information Processing, University of Veterinary Medicine Hannover, Foundation, Bünteweg 2, D-30559 Hanover, Germany; Karl.rohn@tiho-hannover.de; 6 Institute of Food Quality and Food Safety, University of Veterinary Medicine Hannover, Foundation, Bischofsholer Damm 15, D-30173 Hanover, Germany; Johanna.a.popp@gmx.de

**Keywords:** methionine, broiler, reduced crude protein, slaughter characteristics

## Abstract

**Simple Summary:**

The increasing public demand for efficient, sustainably produced poultry meat with simultaneous optimization of animal welfare is calling for amendments in meat production. A starting point to meet this demand consists of complete diets for broilers with reduced crude protein content, supplemented with higher amounts of crystalline amino acids. This ensures sufficient nutritional supply and decreased nitrogen excretion for environmental relief. To optimize the performance and health, despite the drastically reduced protein content (~17.5% finisher diet), the present study compared different methionine sources (methionine hydroxy analog, DL-methionine, and L-methionine) in slightly higher dosages than found in practice, in comparison to a diet meeting the methionine requirements using methionine hydroxy analog. Methionine is the first limiting amino acid and has a significant influence on the health and growth of the animals. The results of the feeding trial showed no differences concerning general health, ileal histology, and performance data of broilers before slaughter. However, the composition of the breast meat differed significantly (highest protein content and lowest fat content in the L-methionine group), and thus provides the impetus for further research.

**Abstract:**

Sustainably produced poultry meat with consideration of animal health poses a challenge for broiler production. Low protein diets with high amounts of synthetic amino acids (AAs) like methionine (Met) are the consequence. In a five-week feeding trial, 360 broilers (Ross 308) assigned to four feeding groups were offered protein-reduced complete diets (starter: 20% crude protein (CP); grower: 18.5% CP; finisher: 17.5% CP), supplemented with essential AAs. The “MHA” group received DL-2-hydroxy-4-(methylthio) butanoic acid (DL-HMTBA; trade name: MHA^®^), groups “L” and “DL” the respective Met source in equivalent concentrations each exceeding the nutritional recommendations. “R-MHA” (“R” for “reduced”) received the minimum required level (using MHA as Met source). Performance exceeded performance goals without differences between the groups. The average feed conversion ratio (FCR) amounted to 1.35. The carcass/body weight ratio of R-MHA was significantly lower (0.782) compared to DL (0.808) and L (0.809). Breast meat of R-MHA contained significantly more fat (144 g/kg dry matter (DM)) compared to L (104 g/kg DM) and significantly lower CP content (R-MHA: 838 g/kg DM; L: 875 g/kg DM). The results indicated possible improvement in slaughter yield by protein-reduced diets supplemented with L-Met, thus recommending further research focusing on the Met influence.

## 1. Introduction

The demand for poultry meat has been growing globally for years. Due to increments in intensive poultry production and the resulting negative effects on the environment [1,2,3], search for the best environmentally-friendly management practice is more relevant than ever. Not only husbandry of the animals itself but also the use of excreta as fertilizer is a problem (high nitrate load on soil). This is also the case in Germany, which is reflected, for example, in the discussion about the tightening of the fertilizer ordinance and the subsequent protests by farmers [4]. The solution to reducing nitrate excretion of animals consists of a significant reduction of the protein content in the feed with simultaneously increased and balanced use of synthetic amino acids (AAs). For instance, in an analysis of Kebreab et al. [5], it was also possible to reduce the greenhouse gas emission of broilers by 54% (in a model based on European conditions) by using a diet with “specialty feed ingredients” (AAs and phytase), compared to an unsupplemented diet.

The present study concentrated, besides the protein reduction in feed, which was already successful in a first trial (protein reduction by 2%; [6]), now on the optimization of the amino acid supply of broilers. More precisely, on the effectiveness of the various Met sources, it is the first limiting AA [7,8] and of great relevance to birds. Met is a sulfur-containing AA and present in natural L form [9]. It contributes to the performance [10], health, and feather growth of the animals, being involved in many metabolic processes, e.g., in protein synthesis [11,12,13], where it is essential, besides structuring of the proteins, for the initialization of translation [14] and thus contributes significantly to the development of the body protein (and, therefore, breast muscle) of the animals [15,16]. As a sulfurous AA, Met serves, inter alia, as a precursor of cysteine (feather growth) and bioactive compounds, such as taurine and glutathione (GSH) [17]. The latter is an antioxidant compound, which is increased by Met and reduces oxidative stress [18,19,20]. By decreasing the activity of fatty acid synthase (lipogenesis) and elevating the hormone-sensitive lipase activity (lipolysis), Met is also capable of regulating the body fat content [21]. All aforementioned functions, when combined, do not only affect health and growth [22,23,24] but ultimately lead to an improved slaughter yield through a higher breast meat yield [23,25] with simultaneously reduced fat content [26,27,28].

Met deficiency has significant negative effects, such as a reduced feed conversion ratio (FCR) in broilers, which results in retarded growth [29,30] and an increased abdominal fat proportion, as Moran et al. [31] noted in week 6 of fattening. Such deficiencies would occur if a corn/soy-based diet was used exclusively, as it contains only a limited amount of L-Met.

Due to the additional elimination of animal products in the feed (e.g., feather meal), the animals are not sufficiently supplied with cysteine as well, so synthetic Met needs to be supplemented. However, L-Met was not commercially available for animal production until it’s registration as a feed additive in 2014 (Commission Implementing Regulation (EU) No 852/2014 of 5 August 2014). Therefore, the racemate DL-Met or MHA (actually DL-2-hydroxy-4-(methylthio)butanoic acid (DL-HMTBA), but in the following, the short trade name MHA is applied) was mainly used, which has a biological availability of 99% or 84%, respectively [32,33]. 

In order for these analogs to be available for the metabolism, they must be converted into the directly metabolizable L-Met, after being absorbed in the small intestine [12]. The D-isomer of DL-Met is first converted in liver and kidney by d-amino-oxidase into 2-keto-4 (methylthio) butanoic acid (KMB) and then by transamination into L-Met. MHA, on the other hand, has to be oxidized to KMB in two different enzymatic systems: first by L-2-hydroxy acid oxidase (L-HAOX; mainly located in hepatic and renal peroxisomes) and by D-2-hydroxy acid dehydrogenase (D-HADH; in the mitochondria of various tissues) [26,34,35,36].

There is controversy in studies, confirming the sufficiency of these analogs (DL-Met, MHA) [37,38]. It is assumed that supplementing L-Met leads to a better efficacy because it could be used directly by the intestinal cells, without prior enzymatic conversion in the liver and kidney, as is required for DL-Met and MHA [24]. In particular, young animals, in which the expression of d-amino acid oxidase just begins and increases moderately [39], are insufficiently supplied with Met. Having a direct effect of L-Met in the intestinal cells, without metabolization through the enzyme, would lead to a better redox status and an improved gastrointestinal development and growth performance of young broilers [24]. 

Due to its important role, Met is generally used in practice above the recommended dose range [6,40,41]. In the present study, not only the Met content but also the concentration of the remaining essential AAs is proportionally elevated due to the reduced crude protein (CP) content. Finally, the hypothesis of this trial was to further optimize the strongly protein-reduced feed with respect to the AA composition by comparing the already described positive attributes of L-Met with other Met sources and to verify the influence on performance and slaughter yield.

## 2. Materials and Methods

The experiment took place, conforming to German regulations. The Animal Welfare Officer of the University of Veterinary Medicine Hannover, Germany (reference: TVT-2018-V-102), approved the experiments. Since no interventions had been carried out on the broilers before dissection, no notification or approval in accordance with the *Animal Protection Act* (Section 7, paragraph 2, sentence 3) was required for this experiment. Two dissections were carried out: the first one on day 7 and the second on day 35 so that the number of animals per box was reduced to 10 after the first week of the trial. For the exclusive use of their organs or tissues for scientific purposes, the animals were killed in accordance with Section 4, paragraph 3 of the *Animal Protection Act*.

### 2.1. Animals, Housing, Experimental Design, and Feeding Concept

For the trial, a common line for fattening of broilers was used (as hatched; ROSS 308; BWE-Brüterei Weser-Ems GmbH & Co. KG; Visbek-Rechterfeld, Germany). The 360 one-day-old broiler chickens of both sexes were divided into four groups (n = 4: MHA; DL; L; R-MHA; with six replicates each; 15 animals per replicate). During the first two weeks, animals from a reserve group replaced impaired or deceased broilers. Thereafter, no animal was replaced anymore. The animals were randomly separated into 24 boxes (1.20 × 0.80 m; AviMax—modified, Big Dutchman International GmbH, Vechta-Calveslage, Germany) at the start of the experiment. The four feeding groups with their replicates were evenly distributed in the stable so that the box location had no influence on the experimental results.

In each box, 1 kg of wood shavings (GOLDSPAN^®^, Goldspan GmbH & Co. KG, Goldenstedt, Germany) served as litter, which covered the floor with at least 1 cm. One side of each box served as a scratching area, optically divided by the drinking line (Big Dutchman International GmbH, Vechta, Germany), with two drinking nipples per box, from the feeding area, equipped with one hanging-type feeder (Klaus Gritsteinwerk GmbH & Co. KG, Bünde, Germany). Ventilation of the stable was provided by a vacuum ventilation system, which was installed on both long sides of the stable above the boxes. The environmental temperature was controlled daily and kept at an adequate range through space heating, and the red lamps above each compartment or additional ventilation (switched on at temperatures above 29 °C) as the weather became hotter during the summer. 

The lighting in the barn was continuous during the first three days, followed by a photoperiod of 16 h (05:00 to 21:00) and 8 h of darkness with dimmed night lighting until the end of the experiment.

Feed was offered ad libitum. In total, four different feeding approaches were prepared, containing four different starter diets, four grower diets, and four finisher diets. All diets were pelleted. In order to maximize the comparability of the final diets, the same supplementary feed (Best 3 Geflügelernährung GmbH, Twistringen, Germany) was used at 80% in all 12 diets. It contained vitamins, minerals, and coccidiostats (narasin/nicarbacin). To complete the respective diets, the remaining 20% were supplemented with equal proportions of soybean meal, AAs (arginine, isoleucine, lysine-HCL, threonine, and valine) and minerals, such as limestone, monocalcium phosphate, salt, sodium bicarbonate (see Appendix A Table A1). Basically, the only difference between the diets was the corn content (to balance the various Met contents) and the Met source (Table 1).

The three feeds adapted to the respective growth phase were fed to all groups in the following periods: starter: d 1–7, grower: d 8–14, finisher: d 15–35. The animals received a complete diet with a protein content oriented towards the protein levels in a previous experiment [6], which had been found to be optimal, and a constant arginine to lysine ratio (calculated CP contents in present trial: starter: 20.1% CP; grower: 18.7% CP; finisher: 18.2% CP; arginine to lysine ratio of approx. 115:100). Table 2 shows the calculated, intended concentrations of the feed ingredients (bioavailability of MHA (84%) included). Based on the excellent results of the mentioned trial [6] with protein-reduced feed, where a reduction of CP (18% in finisher diet) with carefully balanced AAs led to higher body weights (BWs) and significantly decreased N excretion compared to the control group (20% CP in finisher diet), a similarly high content of Met was applied (previous trial, calculated values (88% dry matter (DM)): starter: 6.02 Met g/kg diet; grower: 5.58 Met g/kg diet; finisher: 5.47 Met g/kg diet). The first group, referred to as the control group, received MHA as the only source of added Met. In the following two groups, the added Met sources were different (DL-Met, L-Met). For each of these three groups, the following Met contents were defined (88% DM): starter: 6.02 g/kg diet; grower: 5.81 g/kg diet; finisher: 5.74 g/kg diet (MHAs metabolic availability of 84% was taken into account). The remaining experimental diet showed a lower Met concentration using MHA diet (88% DM): starter: 4.98 g/kg diet; grower: 4.49 g/kg diet; finisher: 3.78 g/kg diet (MHAs metabolic availability of 84% was taken into account). These concentrations exceed the ones recommended by GfE (Gesellschaft für Ernährungsphysiologie e.V.) [42] or NRC (National Research Council) [43] (GfE (88% DM): starter: 3.96 g/kg diet; grower: 3.43 g/kg diet; finisher: 3.43 g/kg diet; NRC (88% DM): starter: 4.93 g/kg diet; grower: 3.70 g/kg diet; finisher: 3.70 g/kg diet).

Essential AA levels were equal in all diets by adding the following crystalline AAs (in addition to Met) in different concentrations: lysine, threonine, valine, isoleucine, arginine (see Table 2 and Table 3, respectively). Due to the addition of MHA, the analysis of Met was performed in two steps, whereby MHA had to be investigated separately in an external laboratory (LUFA-ITL GmbH, AGROLAB Group, 24,107 Kiel, Germany).

### 2.2. Measurements

#### 2.2.1. Technical Performance

Measuring the individual body weight (BW) was done once a week on the same weekday. Exceptions were the first and the last week, in which the interval had to be shortened by one day for technical reasons (six days). At the box level, which corresponds to the replicate level, the feed intakes (FI), water intakes (WI), as well as the animal losses had been determined. Additionally, conspicuous behavior deviating from regular one (like sand bathing, scratching, etc.) was documented. The calculation of the FCR was based on the collected feed intake and body mass increase at the box level.

#### 2.2.2. Litter Sampling

To measure the DM content of the litter, samples were taken weekly at three defined spots along a diagonal line through each box. A cup was used (5 cm diameter) to punch out a sample from the full depth of the bedding, at each collection point. Additionally, the nitrogen (N) content of the samples was tested during the first and last weeks, respectively. In contrast, the samples of the last week were obtained as follows: At the end of the experiment, the bedding material of all boxes was collected separately. To obtain a uniform sample, 10 kg from each box bedding was separated and homogenized (rotating machine). From this material, an aliquot was taken and used for the last DM and N analysis.

#### 2.2.3. Footpad Dermatitis (FPD) Scoring Criteria

The footpads of the animals were scored with a scoring system from 0 to 7 designed by Mayne et al. [44]: score 0 stands for healthy skin with no swelling or redness, whereas score 7 represents a footpad, which is more than 50% necrotic [6]. Only the dried central plantar of footpads was evaluated.

Scoring footpads, as the essential welfare indicator in practice, was carried out weekly. If necessary, the feet were carefully washed with a wet cloth to remove slightly adhering debris and to facilitate the examination. 

#### 2.2.4. Dissection

The first dissection took place after seven days, where 120 animals (n = 30 per group) were examined; the second one after 35 days, where 72 broilers were dissected (n = 18 per group). In both dissections, a percussive blow to the head was used as anesthesia or rather stunning method in accordance with Annex I of Council Regulation (EC) No. 1099/2009, Chapter I, Methods, Table 1—Mechanical methods, No. 6, followed by bleeding the animals. Samples of the ileum were taken for histological investigations by opening the body cavity and lifting the sternum. 

#### 2.2.5. Histological Investigations

In order to determine the size of the villi and crypts in the ileum, as established indicators of intestinal health [45,46,47], the histological samples were processed as follows: During the dissection, a tissue sample of approximately 1 cm^2^ was removed from the ileum (halfway between the diverticulum and the cranial part of the cecum) and fixed in 4% formaldehyde for 48 h. After fixation, tissue samples were embedded in paraffin using standard techniques [48]. For histological evaluation, 4 µm sections of all samples were stained with hematoxylin and eosin (HE) using established protocols [48]. The villus height was measured from the tip of the villi to the villus crypt junction; villus width was measured at the base of the villus above the villus crypt junction; the depth of the crypts of Lieberkuhn was measured from the villus crypt junction to the basal lamina of the crypts (just above the *Lamina muscularis mucosae*). The relation of the villus height to the depth of the crypt was computed at the end [49]. Measurements were performed using a Zeiss Axioscope (Carl Zeiss Jena GmbH, Jena, Germany).

#### 2.2.6. Analysis of the Breast Meat

In addition to the usual carcass parameters (e.g., carcass weight, carcass/body weight ratio etc.), the breast meat quality traits were analyzed at the Institute for Food Quality and Food Safety (University of Veterinary Medicine Hannover, Foundation; [50]). For this purpose, 24 animals (six per group) were slaughtered at the end of the experiment. Measuring the pH value: using a pH meter (Portamess^®^ Type 911 pH, Knick, Germany), a glass electrode (InLab 427^®^, Mettler-Toledo, Urdorf, Switzerland) and a temperature sensor in combination, measurements were taken at the *Musculi pectorales superficiales* (MPS) 24 h post mortem (p.m.). After the same storage time, the drip loss measurements were carried out. The value of the drip loss was obtained by subtracting the pectoral muscle weight after 24 h and the weight after 48 h storage at 4 °C. Therefore, the respective pectoral muscle piece was dabbed dry and stored cool in a plastic box. With a portable electrical conductivity (EC) meter with two parallel stainless steel electrodes (LF-Star^®^, Matthaeus GmbH, Poettmes, Germany), the EC was measured. Shear force: after determining the boiling loss, the shear force was determined using the Warner–Bratzler shear force method with the Texture Analyzer TA.TXplus (Stable Micro Systems, Surrey, England). On a fresh cut in MPS, the CIE (International Commission on Illumination) color values (L* (lightness), a* (redness), b* (yellowness)) were measured with a colorimeter (Minolta CR 400^®^, Konica Minolta GmbH, Langenhagen, Germany). All parameters were measured three times, from which the mean values were calculated, respectively.

#### 2.2.7. Analysis of Feed and Litter Samples

The various diets were analyzed in accordance with the official methods of the VDLUFA [51]. The DM content was determined mathematically by weighing before and after drying the samples at 103 °C. The crude ash content was detected by weighing the litter samples before and after combustion in the muffle furnace at 600 °C. The soxhlet apparatus delivered the crude fat content using a standard protocol and through washing the samples in dilute acids and alkalis, and the crude fiber content was determined. Moreover, the DUMAS incineration method (Vario Max, Elementar, Hanau, Germany) was applied to deliver the results for the total N content. Sugar and minerals were analyzed by using the Luff–Schoorl method (for sugar) and the atomic absorption spectrometry (for minerals) (Unicam Solaar 116, Thermo, Dreieich, Germany). AAs were analyzed by ion-exchange chromatography (AA analyzer LC 3000, Biotronic, Maintal, Germany). Finally, determining the starch content of the diets was accomplished polarimetrically (Polatronic E, Schmidt und Haensch GmbH & Co., Berlin, Germany). 

### 2.3. Statistical Analysis

The statistical analysis was performed using the Statistical Analysis System for Windows the SAS^®^ Enterprise Guide^®^, version 9.3 (SAS Institute Inc., Cary, NC, USA). For all parameters, mean values, as well as the standard deviation (SD) of the mean, were calculated. Feed intake, water intake, the N content, etc. were analyzed at the box level. For parameters, such as BW, FPD scores, and the histological values, the individual animals were the basis of the calculation. The mean of the FPD scores was determined by forming the mean of the scores of both feet of each animal. In general, a one-way-ANOVA for independent samples and mixed models was performed for repeated parameters, i.e., BW, histological measurements, and the DM and N content in the litter. For parameters, which were not distributed normally, a non-parametric one-way-ANOVA was used. The *p* < 0.05 was the basis of a statistical significance for all statements concerning the results of the analysis. 

## 3. Results

Animal losses were limited to a total of six of 360 animals from the third week onwards (MHA: 0; DL: 1; L: 3; R-MHA: 2). In general, the experiments ran without complications and the use of antibiotics or other therapeutic interventions. Controlling the temperature in the stable was significantly more difficult, due to the high outside temperatures. It was gradually reduced from about 33 °C for the one-day-old birds to about 25.2 °C by day 21, rising again to an average of 30.1 °C by day 35.

### 3.1. Technical Performance

In Table 4, the means of the weekly weighing results are shown. There were no significant differences between the BWs of the animals. The average BW of all groups was 44.91 ± 0.98 g at the beginning and 2346 ± 21.10 g at the end of the trial.

Table 5 summarizes the increase in body mass (average of the as-hatched weight compared to the final BW per group), the average total feed intake per animal and group, and the FCR over the entire experimental period (last data collected on day 34). Comparing the groups, there were no significant differences. In general, the BW-gain was high in all groups (2205 ± 23.80 g), and the average FCR in total was 1.35.

No significant differences were observed concerning the water/feed (w/f) intake ratio per day and animal, except week 3 (see Appendix A Table A2). With a w/f intake ratio of 1.87, the 4th group (R-MHA) showed a greater difference between feed (slightly reduced) and water intake (slightly elevated) compared to the other groups (average 1.76).

### 3.2. Litter Quality and Footpad Dermatitis

At the beginning of the trial, the DM content of the litter was 919 g/kg DM. During the trial, there were no statistical differences in DM content between the groups (Table 6). This led to no differences in the health of the footpads of the animals, except in the first week, where R-MHA had a significantly lower score than L. However, it must be considered that scores below one can be neglected. The footpad health with an average score of 0.97 (end of the trial) was very good overall.

Analyzing the N content and the DM content in the litter (week 1 and at the end of the trial) showed no significant differences. Regardless of the *p*-value, the MHA-diets (first and last group) had the wettest litter at the first and the last week. Nevertheless, the N contents were almost identical in all groups (Table 7). In general, the DM content declined by 20%, and the N content increased by 201%.

### 3.3. Results of Dissections

The carcass/body weight ratio showed significant differences (Table 8): R-MHA had the lowest ratio (0.782) compared to DL (0.808) and L (0.809), meaning that carcasses of R-MHA had less usable body mass.

Analyzing the composition of breast meat (Table 9) revealed that R-MHA had a significantly higher fat content (144 g/kg DM), especially compared to L (104 g/kg DM), significantly lower CP content (R-MHA: 838 g/kg DM; L: 875 g/kg DM) and phosphorus content (R-MHA: 8.79 g/kg DM; L: 9.48 g/kg DM).

The analyses of the breast meat conducted by the Institute for Food Quality and Food Safety showed no significant differences whatsoever (Table 10).

### 3.4. Histological Investigations

Neither in the first nor the second round of dissections were significant differences detectable concerning the sizes of the measured villi (Table 11), although it appeared that the villus height of the L- and the R-MHA group was greater than the other two groups at the end of the trial. In the samples of the second dissection, the longest villi tended to have the deepest crypts, which again did not show in the height/crypt depth ratio.

**Figure 1:**
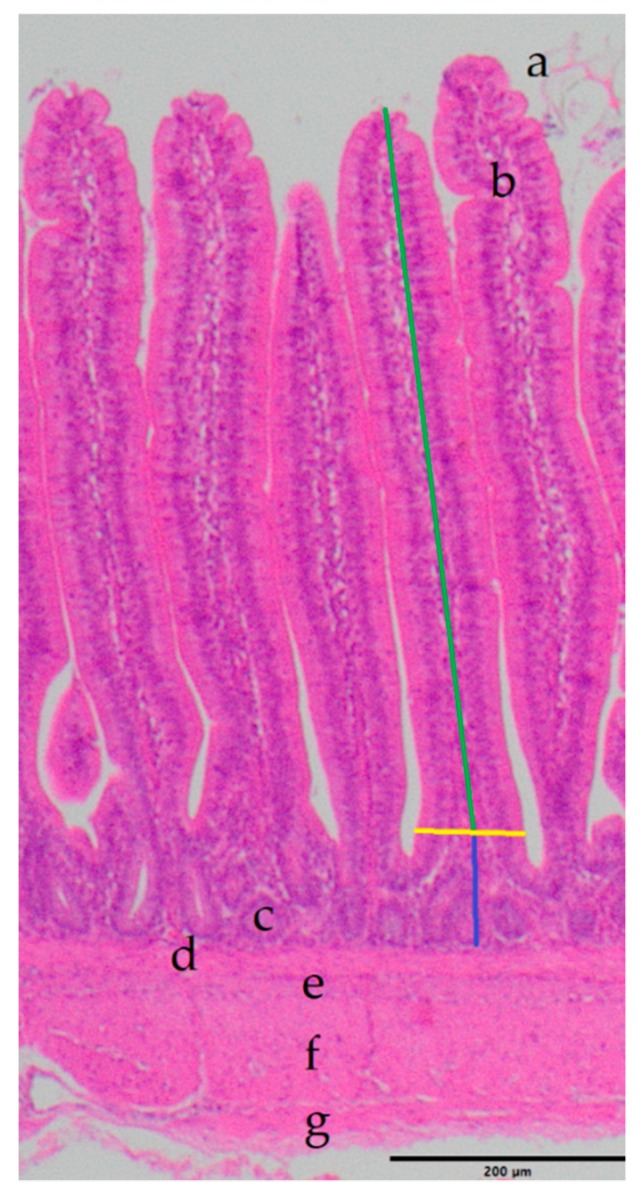
Example image: HE staining from the ileal intestinal wall, measuring lines included. Scale bar = 200 µm. Green: height; yellow: width; blue: crypt depth; a = Villus intestinalis, b = Lamina propria mucosae, c = Lieberkuhn crypt, d = Lamina muscularis mucosae, e = Tela submucosa, f = Tunica muscularis, g = Tunica serosa.

The intestinal wall of the ileum showed no alterations (Figure 1).

## 4. Discussion

There were no incidents during the implementation of the project. Animal losses were very low (1.66% mortality) from day 14 onwards. The performance parameters exceeded the performance goals of the breeding company [52]. In general, the BW-gain was higher in all groups (2205 ± 23.80 g) compared to the Ross 308 performance objectives (2019), where the average BW-gain is 2095 g (weight of day 1 subtracted from the final weight, respectively). The final average BW of all groups was 2346 g and excelled the guidelines of the breeding organization (2235 g) at day 35 by 4.73%. The total FCR (1.35) after 34 days was lower compared to the performance goals of Aviagen (1.46) and to the results of the previous trial (1.47) [6] (values also corrected by the starter weight and taking into account that the animals had to be weighed after six instead of seven days in week 1 and 5). Contrary to the preceding trial [6] under the same conditions, the average feed intake was lower, resulting in an improved FCR. The reduced feed intake was probably due to the prevailing high outside temperatures during the experimental phase. Teeter et al. [53] already reported that high temperatures decreased the FI and the BWG, and this was confirmed, inter alia, by a more recent study conducted by Abu-Dieyeh [54], who observed lower BWs due to heat exposure and a significantly lower FCR. In addition, a study by Bonnet et al. [55] found a decrease in nutrient digestibility (e.g., protein, fat, and starch) when broilers were fed ad libitum at 32 °C. In the present study, the BWG was not affected, indicating that the broilers were not exposed to severe heat stress. In addition, some studies have shown that reduced feed intake or feed restriction increases the passage time of ingesta and thus increases digestibility [56,57,58].

Scoring the footpads was carried out as an essential welfare indicator and to verify whether the different Met sources influence the pathogenesis of FPD. As Chavez and Kratzer found out, MHA is supposed to be less effective in preventing FPD [59]. The possible influence of the different diets on the footpad health did not become evident here, as the impact on the footpads was already low owing, inter alia, to the beneficial husbandry conditions. The FPD score in all groups was ≤1 at the end of the study. These positive results are also attributed to the good litter quality due to the CP-reduced diets, as already postulated by Shao et al. [49] on CP-reduced diets. In addition, the aforementioned study [6] confirmed, as a result of a CP reduction (17% CP finisher diet), that the litter material was significantly drier compared to the control feed (20% CP finisher diet) and, therefore, beneficial for footpad health.

Concerning the N content in the litter, there were no differences between the groups. However, the general N excretion of the animals could be further reduced. Also, in comparison to the preceding study [6] (N content in the litter, end of the trial: 20% CP: 51.20 g/kg; 18% CP: 46.20 g/kg), the N excretion was decreased by 11%, with a continuously advanced performance of the animals. In general, N excretion can be influenced by the CP content, as Blair et al. [60] discovered with a diet containing 18% CP and a resulting lower N excretion by 10%–27%. Nevertheless, without carefully balanced supplementation with crystalline AAs, the CP content and, thus, the N excretion cannot be reduced without performance losses [61]. The two aforementioned parameters (FPD score and N content) clearly confirmed the positive influence on health and the reduction of the environmental impact of N.

As shown in Table 2, the Met levels (finisher diet) in all groups (mean of MHA, DL, L: 5.74 g/kg Met, 88% DM; R-MHA: 3.78 g/kg Met, 88% DM) were higher than recommended by NRC in 1994 (3.70 g/kg Met, 88% DM) and by GfE (3.43 g/kg Met, 88% DM). Higher Met values (in terms of performance parameters) have proven beneficial in numerous studies [23,62]. Already decades ago, e.g., a study in 1964 [63] postulated that a supply of 0.63% Met (finisher diet) maximized the weight gains of the animals. In this previous study, neither the N excretion nor the health characteristics of the animals were taken into account. Further studies, carried out in the 1990s confirmed that performance (BWG and FCR) improved with a Met supply above the recommendation [64,65,66]. For this reason, Aviagen^®^ publishes Nutrition Specifications for Ross^®^ broilers [40], referring to nowadays animal performance and recommending the following regarding Met: starter 5.6 g/kg diet; grower: 5.1 g/kg diet; finisher: 4.7 g/kg diet. Meaning, the diets (MHA, DL, and L) of the present trial still contain higher dosages of Met (Met content in present trial increased by 7% (starter), 12% (grower), and 4.7% (finisher)), except for R-MHA, where the Met content is lower by 12% (starter), 14% (grower), and 24% (finisher). In addition, the Met concentrations were comparable (moderately higher in present study) to those of Lemme et al. at similar protein contents (Met concentration: starter: 5.3 g/kg diet; grower: 5.1 g/kg diet; finisher: 4.8 g/kg diet), who recently published a study on the impact of a decreasing dietary N content on growth performance and N excretion [41].

The results of the histological examination were used to check whether the different Met sources had an obvious influence on ileal histology. Data for the present study gave no indication of this.

The comparison of the Met values with other studies and guidelines was mainly done with the calculated values, due to variations in the analyzed values (Table 3). These could have been caused by the sensitivity of sulfur-containing AAs (SAA) to oxygen during the analysis. Particularly noticeable were the fluctuations in the feed, where relatively high MHA level was used (i.e., the starter, grower, and finisher feed of the MHA group). It is noteworthy that after repeated analyses, the very same samples always showed a slightly different Met content. Problems in the feed production were excluded (fluctuations also occurred after the new production of the feed). The question, therefore, arises whether MHA can influence the detection of native Met without being detected by the analyses itself. In an essay by Brede et al. [67], the SAA detection problem is addressed. Already in 1975, Hušek [68] described the loss of cysteine and Met in the presence of oxygen during analyses. Also, Obreshkova [69] received a lower SAA and hydroxyl AA content in the analysis than the indicated content due to its high sensitivity to traces of oxygen. The storage of the samples also plays a role in the detection of the AA: as Metz et al. [70] were able to prove, a loss of Met occurs after approx. 24 to 36 h. Since the feed produced was analyzed directly, the influence of incorrect storage could be excluded.

The results of the carcass analysis revealed that the group fed the lowest Met concentration (R-MHA) had a significantly higher fat content (144 g/kg DM), especially compared to L (104 g/kg DM), significantly lower CP content (R-MHA: 838 g/kg DM; L: 875 g/kg DM) and phosphorus content (R-MHA: 8.79 g/kg DM; L: 9.48 g/kg DM). As lean meat is in demand as a protein supplier, the supplementation of L-Met also offers an advantage here. The fact that increased dietary Met levels are beneficial is supported, inter alia, by the findings of Hickling et al. [23], who achieved an increased weight gain, feed efficiency (from three-six weeks), and breast meat yield (at six weeks) through a Met supply of up to 118% of the then recommended NRC value. Bunchasak [12] summarized numerous studies and wrote about the role of dietary Met in poultry production. Several studies in this publication confirm that by raising Met supplementation while maintaining a balanced AA supply, the breast muscle yield increases, whereas the abdominal fat content decreases [71,72,73,74].

The mentioned studies were related to the general volatile increase of Met in the feed (especially increased DL-Met). There were no differences in the comparison of Met sources [75]. However, the results were primarily related to performance parameters, such as final BW and weight gain. A possible explanation for the visible differences in the present study would be the reduced protein content associated with the relatively increased proportion of added Met and its availability. Thus, as explained in the introduction, the facilitated metabolizability of L-methionine [12,26,34,35,36] could provide the advantage here, compared to DL-Met and MHA. According to our hypothesis, the advantage of L-Met might have been more evident due to the prevailing optimal husbandry conditions in this trial, in comparison to the more inhomogeneous husbandry conditions in common practice. This hypothesis should be validated with a larger number of animals and different housing conditions. Then, a carefully calculated addition of L-Met could be recommended in order to improve the quality of slaughter performance, to achieve a higher CP content and a lower fat content in carcasses.

In the present study, the influence of feed on the immune system was not focused on, but it would be worthwhile to continue research in this direction, especially regarding the aforementioned lack of d-amino-oxidase in young broilers.

In summary, even when feeding drastically protein-reduced diets, the animals could still achieve high performance through an optimized AA supply. However, the different Met sources seemed to have no influence on parameters, such as FI and BWG, in the present study, contrary to the parameters of the breast meat analyses, such as a decreased fat content in the pectoral muscle through the use of L-Met (compared to R-MHA). Especially regarding consumers’ increasing desire for low-fat meat, these results are certainly interesting and provide impetus for further research into optimized AA supply with an influence on animal health and resource efficiency.

## 5. Conclusions

Since the trial took place under optimal husbandry conditions, the Met supply was above the assumed demand (finisher diet, difference of MHA, DL, L compared to NRC: 35.54%; MHA, DL, L compared to GfE: 40.24%; difference of R-MHA compared to NRC: 2.12%; R-MHA compared to GfE: 9.26%), and the diets differed only by one AA, it is difficult to expect great differences between the groups. On the other hand, the conditions in practice are continuously improving and thus are becoming more similar to the ones in the trial, so that our findings are actually related to practical conditions. The effects of different Met sources on performance (with already high Met supplementation) and protein reduction leave much to be desired. However, the results of the pectoral muscle analysis are interesting. These provide first indications of significant differences in the composition of the muscle when MHA, L-, and DL-Met (except for MHA in minimum required levels) are used in common dosages. Although the number of samples was limited, the findings provide an opportunity for further research into the influence of Met with a sharply reduced CP content in the feed. If one also considers the significantly lower N excretion, inter alia, due to the strong CP reduction, a remarkable improvement results, concerning the environmental impact of poultry production.

These meticulously balanced, protein-reduced diets, considering the Met availability, comprise interesting benefits when it comes to sustainability in animal production.

## Figures and Tables

**Table 1:** The content of corn and the respective methionine sources in experimental diets.

Item (g/kg; 88% DM)		Starter				Grower				Finisher		
	MHA	DL	L	R-MHA	MHA	DL	L	R-MHA	MHA	DL	L	R-MHA
Corn	85.4	86	86	86.7	122.6	123.2	123.2	124.2	144.4	145	145	146.8
MHA ^1^	7.15	-	-	5.93	6.93	-	-	5.35	6.83	-	-	4.50
DL-Methionine	-	6.02	-	-	-	5.81	-	-	-	5.74	-	-
L-Methioine	-	-	6.02	-	-	-	5.81	-	-	-	5.74	-

^1^ Due to the 84% bioavailability of DL-2-hydroxy-4-(methylthio) butanoic acid (MHA), the corresponding values of the diets DL and L are equivalent. R-MHA: reduced MHA; DM: dry matter.

**Table 2:** Template: Calculated nutrient composition of ingredients in the starter, grower, and finisher diets.

Item [g/kg; 88% DM]		Starter				Grower				Finisher		
	MHA	DL	L	R-MHA	MHA	DL	L	R-MHA	MHA	DL	L	R-MHA
Crude fat (EE)	70.9	71	71	71	80.6	80.6	80.6	80.6	80.2	80.2	80.2	80.3
Crude fiber	23.5	23.5	23.5	23.5	23.1	23.1	23.1	23.2	23.2	23.2	23.2	23.3
Crude protein	202	201	201	201	187	187	187	187	182	182	182	181
Starch	389	389	389	390	411	411	411	412	423	424	424	425
Sugar	42.9	42.9	42.9	42.9	39.7	39.7	39.7	39.8	38.7	38.8	38.8	38.8
Calcium	10.3	10.3	10.3	10.3	7.96	7.96	7.96	7.96	6.42	6.42	6.42	6.42
Phosphorus	6.49	6.50	6.50	6.50	5.84	5.84	5.84	5.84	5.26	5.26	5.26	5.27
Potassium	7.38	7.38	7.38	7.38	6.65	6.65	6.65	6.66	6.42	6.42	6.42	6.43
Arginine	14.3	14.3	14.3	14.3	13.1	13.1	13.1	13.1	12.7	12.7	12.7	12.7
Cysteine	3.24	3.24	3.24	3.24	3.10	3.10	3.10	3.10	3.06	3.06	3.06	3.06
Isoleucine	9.17	9.17	9.17	9.17	8.42	8.42	8.42	8.43	8.01	8.01	8.01	8.02
Leucine	13.6	13.6	13.6	13.7	12.7	12.7	12.7	12.7	12.5	12.5	12.5	12.5
Lysine	12.4	12.4	12.4	12.4	11.4	11.4	11.4	11.4	11	11	11	11
Methionine ^1^	6.01	6.02	6.02	4.98	5.82	5.81	5.81	4.49	5.74	5.74	5.74	3.78
Met + Cys	9.25	9.26	9.26	8.22	8.92	8.91	8.91	7.59	8.80	8.80	8.80	6.84
Phenylalanine	8.55	8.55	8.55	8.56	7.84	7.84	7.84	7.85	7.62	7.62	7.62	7.63
Threonine	8.84	8.85	8.85	8.85	8.22	8.22	8.22	8.22	7.84	7.84	7.84	7.84
Valine	10.1	10.1	10.1	10.1	9.31	9.31	9.31	9.31	8.84	8.84	8.84	8.85
Metabolizable energy AME_N_ [MJ/kg DM] ^2^	12.6	12.6	12.6	12.6	13	13	13	13.1	13.2	13.2	13.2	13.2

^1^ Bioavailability of MHA (84%) taken into account; ^2^ AME_N_ = nitrogen-corrected apparent metabolizable energy; AME_N_ (per kg) = 0.1551 × % crude protein (CP) + 0.3431 × % ether extracts (EE) + 0.1669 × % starch + 0.1301 × % sugar (as sucrose).

**Table 3:** Concentrations of ingredients after chemical analysis in the starter, grower, and finisher diets.

Item [g/kg; 88% DM]		Starter				Grower				Finisher		
	MHA	DL	L	R-MHA	MHA	DL	L	R-MHA	MHA	DL	L	R-MHA
Crude ash	54.4	52.3	51.5	55.3	47.5	46.6	49.2	49	43	44.6	43	44.6
Crude fat (EE)	67.2	69.3	68.1	71.6	78.3	76.2	78.3	80.1	80.3	78.3	80.3	78.3
Crude fiber	21	21.7	21.6	22.4	21.4	22.9	22.4	24.2	20.3	20.1	20.3	20.1
Crude protein	194	197	196	198	186	186	184	182	178	178	178	178
Nitrogen free extract ^1^	543	540	543	533	547	548	546	545	558	559	558	559
Starch	386	394	392	381	402	412	396	408	421	417	412	407
Sugar	44.8	45.3	45.1	46.6	41.5	43.3	43.7	41.7	39.6	39.4	39.2	39
Calcium	10	9.66	10	10.7	8.91	7.22	9.62	8.93	6.68	6.34	7.42	7.77
Phosphorus	5.89	5.61	5.84	6.08	5.33	5.65	6.10	5.92	4.93	5	5.25	5.45
Potassium	7.33	7.05	7.09	6.94	6.55	6.68	6.64	6.47	6.43	6.50	6.54	6.65
Arginine	13.6	13.4	14.1	14.7	12.8	13.4	13	13.4	12.5	12.7	12.4	12.6
Cysteine	3.16	3.19	3.07	3.14	2.91	3.10	3.36	3.18	2.88	2.94	2.86	2.92
Histidine	4.39	4.46	4.51	4.65	4.09	4.23	3.98	4.30	4.25	4.10	3.89	4
Isoleucine	8.89	8.88	9.26	9.56	8.10	8.83	8.40	8.74	8.37	8.20	7.70	7.98
Leucine	13	12.9	13.5	14	12.4	13.1	12.3	13.2	13.1	12.8	12	12.4
Lysine	11.8	11.6	12.2	12.7	11.3	11.7	11.4	11.9	11.1	11.1	10.7	10.9
Met (native)	2.41	5.73	5.42	2.94	2.49	5.35	5.66	3.18	2.22	5.08	5.26	2.76
MHA ^2^	2.96	-	-	1.91	2.68	-	-	1.78	2.77	-	-	1.53
MHA (b.a. 84%) ^3^	2.49	-	-	1.60	2.25	-	-	1.50	2.33	-	-	1.29
Total Met incl. MHA (b.a. 84%) ^3^	4.90	5.64	5.4	4.54	4.74	5.35	5.66	4.68	4.55	5.05	5.3	4.05
Met + Cys	8.06	8.83	8.47	7.68	7.65	8.45	9.06	7.86	7.43	7.99	8.16	6.97
Phenylalanine	8.30	8.21	8.56	8.91	7.77	8.13	7.72	8.29	8.11	7.80	7.49	7.56
Threonine	8.32	8.57	8.98	8.56	7.64	7.89	7.96	7.92	7.50	7.98	7.80	7.71
Valine	9.94	10	10.3	10.6	9.37	9.98	9.30	9.83	9.32	9.16	8.77	8.71
Metabolizable energy AME_N_ [MJ/kg DM] ^4^	12.3	12.6	12.5	12.5	12.8	12.9	12.7	12.9	12.9	12.9	12.9	12.8

^1^ Nitrogen-free extract = DM − (crude ash + crude fat + crude fiber + crude protein); ^2^ MHA^®^ analyzed by LUFA-ITL GmbH, AGROLAB Group, 24,107 Kiel, Germany; ^3^ b.a. = bioavailability; ^4^ AME_N_ = nitrogen-corrected apparent metabolizable energy; AME_N_ (per kg) = 0.1551 × % CP + 0.3431 × % ether extracts (EE) + 0.1669 × % starch + 0.1301 × % sugar (as sucrose).

**Table 4:** Average body weight (BW) during the trial (mean ± standard deviation (SD)).

Item	Body Weight [in g, End of Week]			
	MHA	DL	L	R-MHA
Week 1 ^1^	171 ± 13.1	172 ± 15.3	170 ± 13.7	171 ± 14
Week 2	543 ± 47.5	552 ± 45	549 ± 44.5	545 ± 50.9
Week 3	1002 ± 159	1027 ± 99.4	1031 ± 97.8	1030 ± 126
Week 4	1676 ± 203	1682 ± 181	1686 ± 174	1705 ± 192
Week 5 ^1^	2228 ± 307	2242 ± 243	2237 ± 241	2283 ± 279

Statistical analysis: one-way ANOVA for independent samples; ^1^ only six days—measurement at the end of days 6 and 34, respectively.

**Table 5:** In total: Mean of body weight (BW) gain, feed intake (FI), and feed conversion ratio (FCR) during the trial (days 1–34; mean ± SD).

Item	MHA	DL	L	R-MHA
BW-gain [in g] ^1^	2184 ± 158	2196 ± 75	2193 ± 81.3	2245 ± 107
FI [in g] ^1^	2985 ± 117	2929 ± 88.5	2952 ± 131	3012 ± 136
FCR ^2^	1.37 ± 0.058	1.33 ± 0.014	1.35 ± 0.029	1.34 ± 0.016

^1^ Univariate ANOVA; ^2^ Wilcoxon Signed-Rank test (not normally distributed).

**Table 6:** DM content of litter material and footpad dermatitis (FPD) scores (mean ± SD).

Item	Dry Matter Litter [in g/kg End of Week]				FPD Scores			
	MHA	DL	L	R-MHA	MHA	DL	L	R-MHA
Week 1 ^1^	801 ± 44.4	841 ± 67.7	823 ± 59.9	799 ± 43.4	0.06 ^A,B^ ± 0.17	0.06 ^A,B^ ± 0.16	0.06 ^B^ ± 0.19	0.02 ^A^ ± 0.09
Week 2	816 ± 20	798 ± 25.5	790 ± 37.6	796 ± 57.5	0.39 ± 0.41	0.38 ± 0.41	0.39 ± 0.39	0.35 ± 0.39
Week 3	732 ± 36.8	724 ± 13.8	726 ± 30.9	709 ± 27	0.81 ± 0.26	0.73 ± 0.35	0.72 ± 0.34	0.70 ± 0.35
Week 4	672 ± 55.4	680 ± 46.9	681 ± 33.5	661 ± 77.7	0.91 ± 0.20	0.93 ± 0.17	0.93 ± 0.17	0.91 ± 0.20
Week 5 ^1^	584 ± 50.3	583 ± 44	590 ± 67.7	574 ± 80.4	0.93 ± 0.17	0.97 ± 0.16	0.98 ± 0.13	0.98 ± 0.09

Statistical analysis: one-way ANOVA for DM content, non-parametric one-way ANOVA for FPD scores; ^1^ only six days—measurement at the end of days 6 and 34, respectively; ^A,B^ averages differ significantly within a column (*p* < 0.05).

**Table 7:** DM of litter and nitrogen content after the first week (day 7) and of total litter at the end (mean ± SD).

Item	DM Litter [in g/kg]				N [in g/kg DM]			
	MHA	DL	L	R-MHA	MHA	DL	L	R-MHA
Week 1 ^1^	801 ± 44.4	841 ± 67.7	823 ± 59.9	799 ± 43.4	13.5 ± 0.80	13.3 ± 1.69	14.4 ± 1.19	13.1 ± 1.17
End	646 ± 53.2 ^1^	654 ± 39.3 ^1^	664 ± 79.2 ^1^	646 ± 111 ^1^	40 ± 0.78 ^2^	42 ± 1.14 ^2^	39.9 ± 0.76 ^2^	41.8 ± 1.77 ^2^

Statistical analysis: one-way ANOVA for independent samples; ^1^ only six days—measurement at the end of days 6 and 34, respectively; ^2^ after homogenizing in a rotating machine.

**Table 8:** Dissection parameter of 72 broilers (obtained at day 35).

Item	MHA	DL	L	R-MHA
BW [in g]	2413 ± 339	2317 ± 285	2407 ± 250	2376 ± 351
Carcass weight [in g]	1943 ± 284	1874 ± 249	1948 ± 199	1860 ± 326
Carcass/BW	0.805 ^a,b^ ± 0.025	0.808 ^a^ ± 0.022	0.809 ^a^ ± 0.011	0.782 ^b^ ± 0.062
Liver weight [in g]	54.7 ± 11.9	51.8 ± 8.92	54.8 ± 9.08	49.8 ± 9.57

Statistical analysis: univariate ANOVA with Least significant difference (LSD) test; ^a,b^ averages differ significantly within a row (*p* < 0.05).

**Table 9:** Analysis of the breast meat of 24 broilers (day 35).

Item [g/kg]	MHA	DL	L	R-MHA
Dry matter ^1^	247 ^a,b^ ± 14.9	238 ^b^ ± 18.1	256 ^a^ ± 5.34	256 ^a,b^ ± 12.3
Crude ash	48.6 ^a,b^ ± 2.02	49.5 ^a^ ± 3.11	49.4 ^a^ ± 1.77	46.5 ^b^ ± 2.22
Crude protein	865 ^a,b^ ± 22.3	848 ^a,b^ ± 28.2	875 ^a^ ± 33.6	838 ^b^ ± 24.7
Crude fat	114 ^a,b^ ± 20.9	126 ^a,b^ ± 28.6	104 ^b^ ± 30.7	144 ^a^ ± 22.3
P	9.28 ^a,b^ ± 0.44	9.19 ^a,b^ ± 0.41	9.48 ^a^ ± 0.36	8.97 ^b^ ± 0.38

^1^ Non-parametric, one-way-ANOVA for DM; remaining parameters: univariate ANOVA with LSD-test; ^a,b^ averages differ significantly within a row (*p* < 0.05).

**Table 10:** Parameters of slaughter yield (n = 24 broilers), analyzed by the Institute for Food Quality and Food Safety (day 35).

Item	MHA	DL	L	R-MHA
Carcass weight [in g] ^1^	2512 ± 238	2439 ± 220	2292 ± 288	2323 ± 361
Weight both thighs [in g] ^2^	490 ± 40.5	491 ± 53.6	454 ± 48.2	473 ± 64.6
Chest muscle (both) [in g] ^1^	628 ± 83.5	610 ± 78.5	562 ± 86.1	566 ± 93.8
Chest muscle after 24 h (left) [in g] ^1^	254 ± 37.3	252 ± 34	231 ± 39.8	224 ± 36.9
Chest muscle after 24 h (right) [in g] ^2^	257 ± 35.9	250 ± 33.8	234 ± 40.3	236 ± 45.4
Drip loss after 72 h [in g] ^2^	251 ± 33.4	245 ± 33.1	229 ± 38.9	232 ± 44.4
Drip loss after 72 h [%] ^1^	2.29 ± 0.80	1.88 ± 0.30	2.38 ± 1.33	1.68 ± 0.70
Thawing weight [in g] ^2^	237 ± 32.7	232 ± 33.5	219 ± 41.4	222 ± 40.7
Thawing loss [in g] ^1^	5.42 ± 0.70	5.38 ± 1.44	4.29 ± 2.44	4.08 ± 1.70
Cooking yield [in g] ^1^	190 ± 25	186 ± 18.2	175 ± 31	184 ± 34.9
Cooking loss [in g] ^1^	19.9 ± 3.36	19.5 ± 4.72	19.9 ± 1.81	16.9 ± 3.17
Conductivity after 24 h [S/m] ^1^	5.83 ± 2.14	5.21 ± 1.39	4.46 ± 1.01	4.62 ± 1.51
Mean pH value after 24 h ^1^	5.79 ± 0.10	5.78 ± 0.13	5.75 ± 0.11	5.81 ± 0.23
Shear force [in g] ^2^	25.4 ± 7.31	21.1 ± 7.23	22.6 ± 10.9	22.1 ± 7.95
L *^,3^	55.7 ± 2.37	54.7 ± 4.26	55.5 ± 1.69	54.4 ± 2.09
a *^,3^	3.63 ± 1.08	4.27 ± 1.02	3.38 ± 0.98	4.40 ± 1.20
b *^,3^	8.41 ± 1.53	8.81 ± 2.36	7.26 ± 1.20	8.81 ± 2.71

^1^ univariate ANOVA with LSD-test; ^2^ Wilcoxon Signed-Rank test (not normally distributed); ^3^ CIE color values (L* (lightness), a* (redness), b* (yellowness)).

**Table 11:** Average villus height *, width, crypt depth, and height/crypt depth ratio of the ileum (mean ± SD).

Item [in µm]		Dissection I				Dissection II		
	MHA	DL	L	R-MHA	MHA	DL	L	R-MHA
Villus height	420 ± 66.9	405 ± 78.3	418 ± 81.2	407 ± 63.3	557 ± 111	565 ± 103	595 ± 146	594 ± 121
Villus width	102 ± 16.4	99.6 ± 25.7	97.9 ± 17.4	104 ± 17.1	94.1 ± 18	97.4 ± 13.5	94.6 ± 14.7	90.8 ± 12.7
Crypt depth	133 ± 17.6	131 ± 18.9	130 ± 15.8	135 ± 21.5	145 ± 21.3	150 ± 19.2	160 ± 24.7	154 ± 25.9
Height/CD *^,1^	3.20 ± 0.543	3.16 ± 0.816	3.24 ± 0.635	3.08 ± 0.651	3.86 ± 0.599	3.80 ± 0.713	3.73 ± 0.803	3.90 ± 0.660

Statistical analysis: one-way ANOVA for independent samples; * Villus height was measured from the tip of the villus to the villus crypt junction; villus width was measured at the base of the villus above the villus crypt junction; depth of the crypts of Lieberkuhn was measured from the villus crypt junction to the basal lamina of the crypts; ^1^ Crypt depth (CD).

## Appendix A

**Table A1:** Ingredient composition of the diets during the whole experimental period.

Parameter [%]		Starter				Grower				Finisher		
	MHA	DL	L	R-MHA	MHA	DL	L	R-MHA	MHA	DL	L	R-MHA
Supplementary feeding stuff ^1^	80	80	80	80	80	80	80	80	80	80	80	80
Corn	8.54	8.60	8.60	8.67	12.3	12.3	12.3	12.4	14.4	14.5	14.5	14.7
Soybean meal	6.20	6.20	6.20	6.20	2.40	2.40	2.40	2.40	1	1	1	1
Plant oil	1.70	1.70	1.70	1.70	2.60	2.60	2.60	2.60	2.50	2.50	2.50	2.50
Limestone	1.05	1.05	1.05	1.05	0.52	0.52	0.52	0.52	0.20	0.20	0.20	0.20
Mono-Ca-phosphate	0.68	0.68	0.68	0.68	0.45	0.45	0.45	0.45	0.20	0.20	0.20	0.20
Salt	0	0	0	0	0	0	0	0	0	0	0	0
Sodium bicarbonate	0.16	0.16	0.16	0.16	0.15	0.15	0.15	0.15	0.13	0.13	0.13	0.13
Arginine	0.29	0.29	0.29	0.29	0.29	0.29	0.29	0.29	0.28	0.28	0.28	0.28
Isoleucine	0.22	0.22	0.22	0.22	0.20	0.20	0.20	0.20	0.18	0.18	0.18	0.18
L-Lysine-HCl	0.36	0.36	0.36	0.36	0.35	0.35	0.35	0.35	0.35	0.35	0.35	0.35
Methionine ^2^	0.37	0.31	0.31	0.24	0.36	0.30	0.30	0.20	0.36	0.30	0.30	0.12
Threonine	0.24	0.24	0.24	0.24	0.24	0.24	0.24	0.24	0.22	0.22	0.22	0.22
Valine	0.20	0.20	0.20	0.20	0.19	0.19	0.19	0.19	0.16	0.16	0.16	0.16

^1^ Containing 53.8% wheat, 24.1% soybean meal, 15% corn, 4.26% plant oil, 1.32% limestone, 0.94% mono-Ca-phosphate, 0.70% formic acid, 0.25% enzyme mixture (phytase, xylanase), 0.25% premix, 0.23% sodium bicarbonate, 0.20% salt, 0.11% lysine, 0.06% betaine (33%); feed additives per kg: 12,500 IU vitamin A, 6250 IU vitamin D3, 43.8 mg Vitamin E, 18.8 mg Cu, 25 mg Fe, 87.5 mg Mn, 62.5 mg Zn, 2.5 mg I, 0.38 mg Se, acetic acid, formic acid, 625 FTU 6-phytase, 3000 U Endo 1,4-ß-xylanase, 62.5 mg narasin, 62.5 mg nicarbacin; ^2^ three different methionine sources resp. decreased methionine concentration (in one case) depending on diet.

**Table A2:** Average of water/feed intake ratio per day and animal.

Item	Group	Week 1 ^1^	Week 2	Week 3	Week 4	Week 5 ^1^
Water/feed ratio per day	MHA	2.10 ± 0.21	2.15 ± 0.20	1.72 ± 0.05 ^A^	1.87 ± 0.09	1.84 ± 0.06
	DL	2.02 ± 0.06	2.14 ± 0.07	1.79 ± 0.05 ^A^	1.89 ± 0.08	1.84 ± 0.08
	L	2.02 ± 0.34	2.21 ± 0.09	1.77 ± 0.08 ^A^	1.88 ± 0.05	1.84 ± 0.03
	R-MHA	2.09 ± 0.24	2.15 ± 0.15	1.87 ± 0.11 ^B^	1.91 ± 0.11	1.82 ± 0.11

Statistical analysis: one-way ANOVA for independent samples; ^1^ only six days—measurement at the end of day 6 and 34, respectively, ^A,B^ averages differ significantly within a column (*p* < 0.05).
